# Supplementary material for: Venomix: a simple bioinformatic pipeline for identifying and characterizing toxin gene candidates from transcriptomic data
Source: PeerJ. 2018 Jul 31;6:e5361. doi: 10.7717/peerj.5361 (PMC6074769; doi:10.7717/peerj.5361)
Supplement: Supplemental Information 3 [file peerj-06-5361-s003.gz › FinalOutput_E-20/C-type_lectin_6/finaltree.pdf]

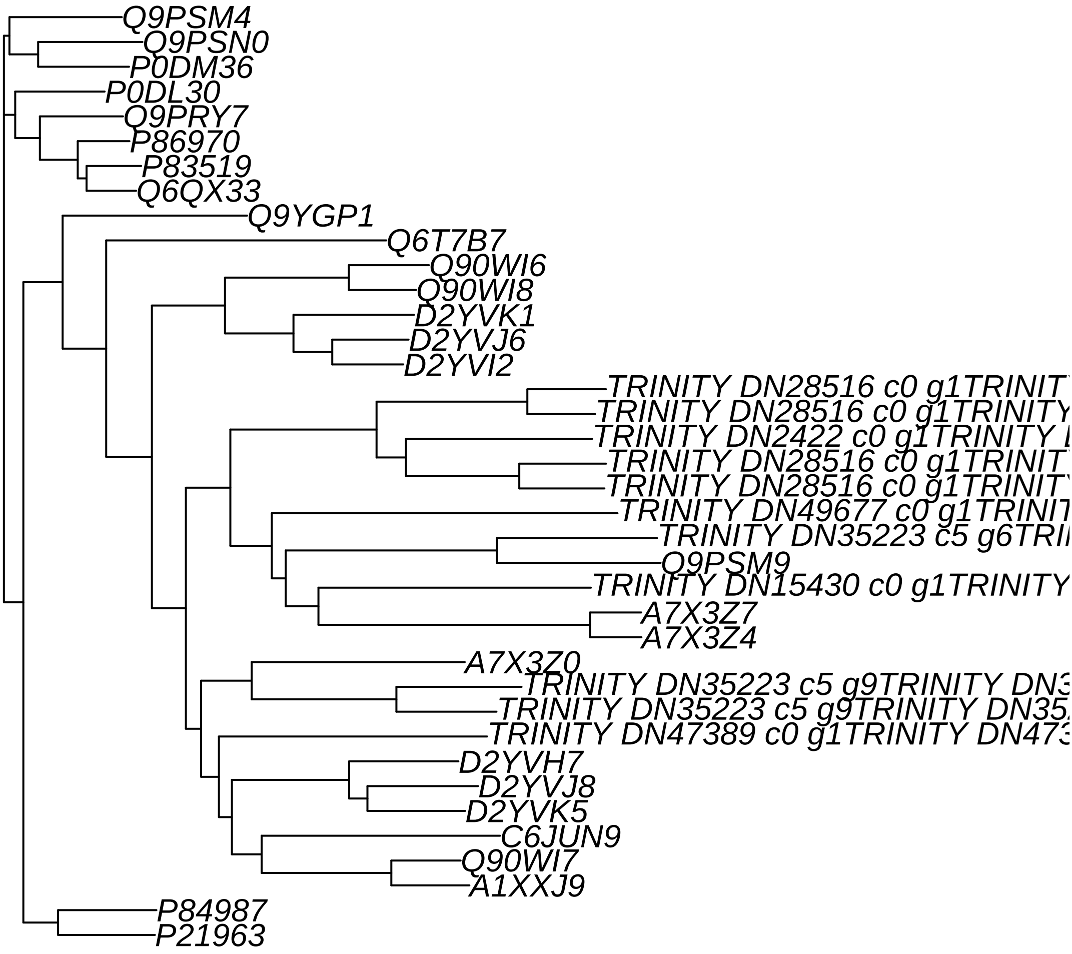
Q9PSM4
Q9PSN0
P0DM36
P0DL30
Q9PRY7
P86970
P83519
Q6QX33
Q9YGP1
Q6T7B7
Q90WI6
Q90WI8
D2YVK1
D2YVJ6
D2YVI2
TRINITY DN28516 c0 g1TRINITY
TRINITY DN28516 c0 g1TRINITY
TRINITY DN2422 c0 g1TRINITY
TRINITY DN28516 c0 g1TRINITY
TRINITY DN28516 c0 g1TRINITY
TRINITY DN49677 c0 g1TRINIT
TRINITY DN35223 c5 g6TRI
Q9PSM9
TRINITY DN15430 c0 g1TRINITY
A7X3Z7
A7X3Z4
A7X3Z0
TRINITY DN35223 c5 g9TRINITY DN3
TRINITY DN35223 c5 g9TRINITY DN35
TRINITY DN47389 c0 g1TRINITY DN473
D2YVH7
D2YVJ8
D2YVK5
C6JUN9
Q90WI7
A1XXJ9
P84987
P21963
